# Surgical repair of a rare sternal origin pectoralis major rupture using a novel mesh-anchor-suture technique: a case report

**DOI:** 10.1093/jscr/rjaf1047

**Published:** 2026-01-07

**Authors:** Hafez Saade, Antoine Saber, Firas Al Hassan, Jihad Boutrous, Joe Abi Akl

**Affiliations:** Department of Orthopedics and Traumatology, Faculty of Medicine, University of Balamand, Dekwaneh Campus, Rond Point Saloumeh, Dekwaneh, PO Box 55251, Sin El Fil, Lebanon; Department of Orthopedics and Traumatology, Faculty of Medicine, University of Balamand, Dekwaneh Campus, Rond Point Saloumeh, Dekwaneh, PO Box 55251, Sin El Fil, Lebanon; Department of Orthopedics and Traumatology, Faculty of Medicine, University of Balamand, Dekwaneh Campus, Rond Point Saloumeh, Dekwaneh, PO Box 55251, Sin El Fil, Lebanon; Department of Surgery, Faculty of Medicine, Lebanese University, Rafic Hariri University Campus, Hadath, Baabda District, PO Box 6573/14, Beirut, Lebanon; Department of Orthopedics and Traumatology, Faculty of Medicine, University of Balamand, Dekwaneh Campus, Rond Point Saloumeh, Dekwaneh, PO Box 55251, Sin El Fil, Lebanon

**Keywords:** pectoralis major rupture, sternal origin, isolated sternal head rupture, repair of pectoralis major, chronic tear with retraction

## Abstract

In this case report, we present a 33-year-old male patient with a rare, chronic, isolated rupture of the sternal head of the pectoralis major (PM) at its sternal origin following a combat sport injury. Managing this case proved challenging due to the chronicity of the injury, which caused significant retraction and suboptimal tissue quality, and the scarcity of available literature regarding such cases. We employed a novel surgical technique using a polypropylene mesh-anchor-suture construct to reinsert the muscle to its sternal origin. Recovery was uneventful, with restoration of near full range of motion and strength by 6 months post-surgery. Our approach to this case demonstrates the feasibility of surgical repair of rare PM ruptures of sternal origin, even with delayed presentation, using modified surgical techniques.

## Introduction

In 1822, Pâtissier recorded the first case of pectoralis major (PM) rupture; a young boy who sustained the injury while lifting a heavy piece of meat from a hook [1] Although rare, rupture of the PM is becoming more common due to an increased interest in sports, fitness, and weightlifting [2]. These injuries are seen more often in young athletic males [3]. Most PM ruptures involve the tendon near its insertion on the humerus, particularly at the musculotendinous junction or the tendon's attachment to the bone, in contrast to ruptures at the sternal origin of the muscle which are very rare. Based on current knowledge, cases involving sternal origin ruptures are scarcely reported with fewer than five cases being mentioned in literature. In this review we describe a novel surgical technique used to repair an isolated PM sternal head rupture at its origin at the sternum in a 33-year-old male using a mesh-anchor-suture construct.

## Case presentation

### Patient description and case history

We present a 33-year-old previously healthy male who visited our clinic for a 2-month history of left PM dysfunction following a combat sports injury. The patient reported sustaining direct trauma to his left chest during training, immediately experiencing sharp pain accompanied by a distinct tearing sensation. Despite recognizing weakness during certain movements, the patient initially attributed these findings to muscle strain. After 2 months of failed conservative management with persistent weakness, the patient sought evaluation at our institution.

Clinical findings prompted further evaluation. However, the patient preferred not to undergo MRI and was therefore, asked to undergo a dynamic ultrasound of the PM which showed a sub-total rupture of the sternal head of the PM at the sternal origin (with only a small portion of the distal origin of the muscle intact) with retraction.

Immediate surgical repair was recommended based on Bodendorfer's evidence supporting early intervention [4], however personal circumstances necessitated an 8-month delay.

### Surgical management

After positioning, scrubbing and draping, dissection was performed in layers until the torn PM was identified and exposed. The retracted muscle was extensively released from adhesions.

**Figure 1:**
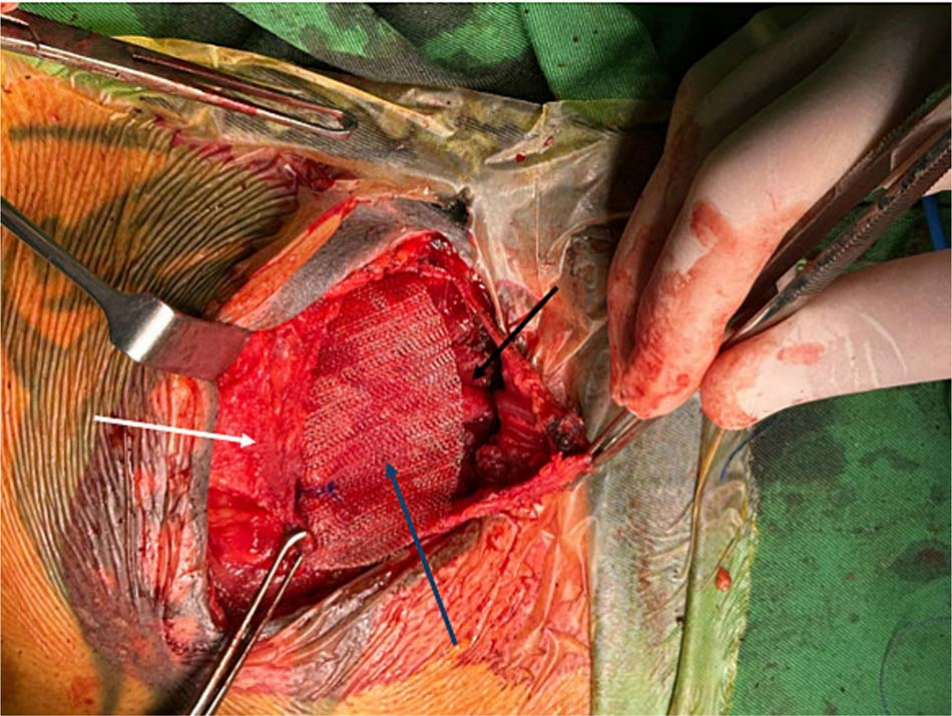
Intraoperative image showing polypropylene mesh (blue arrow) positioned between the PM muscle (black arrow) and the overlying pectoral fascia (white arrow). The sternum is to the right of the image.

**Figure 2:**
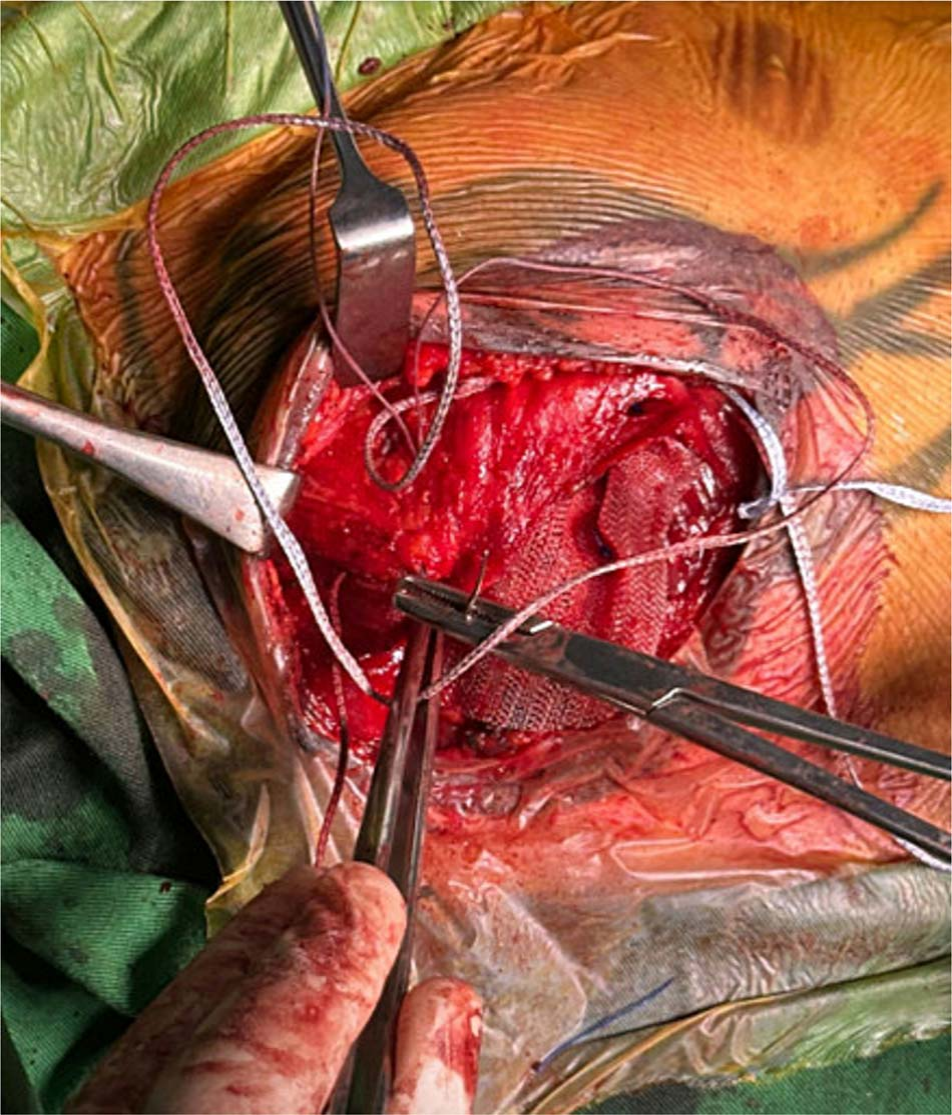
Intraoperative image showing suturing of the PM muscle and mesh to the overlying pectoral fascia.

A polypropylene mesh was trimmed and placed over the PM and beneath the pectoral fascia (Fig. 1). It was secured to both the PM and fascia using non-absorbable 2–0 Prolene sutures (Fig. 2).

**Figure 3:**
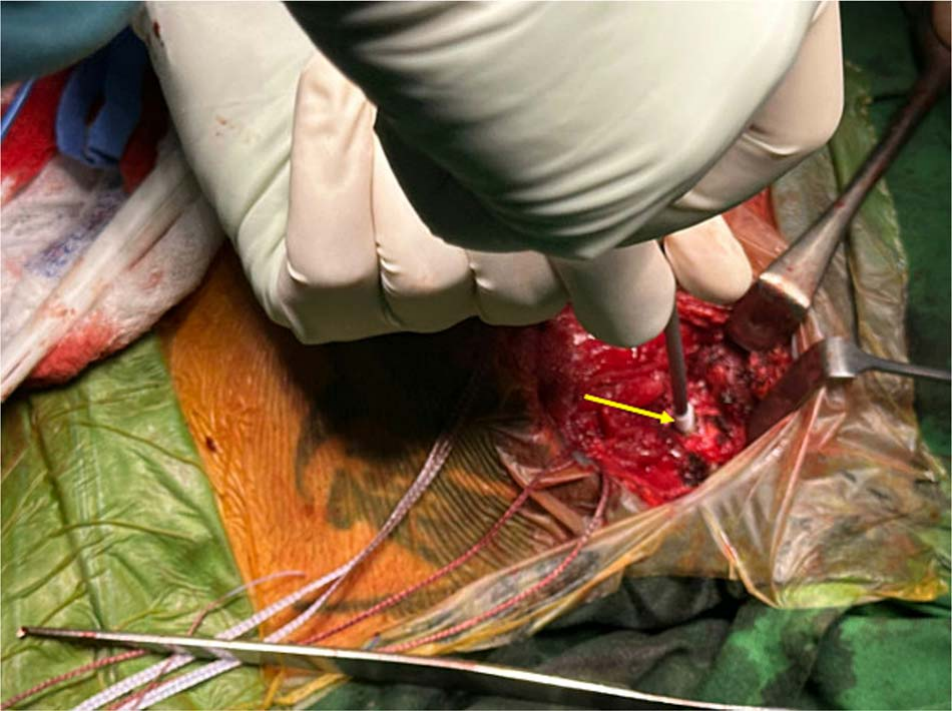
Intraoperative image showing insertion of a knotless suture anchor (arrow) into the sternum.

Three knotless anchors were implanted vertically in the sternum (Fig. 3).

**Figure 4:**
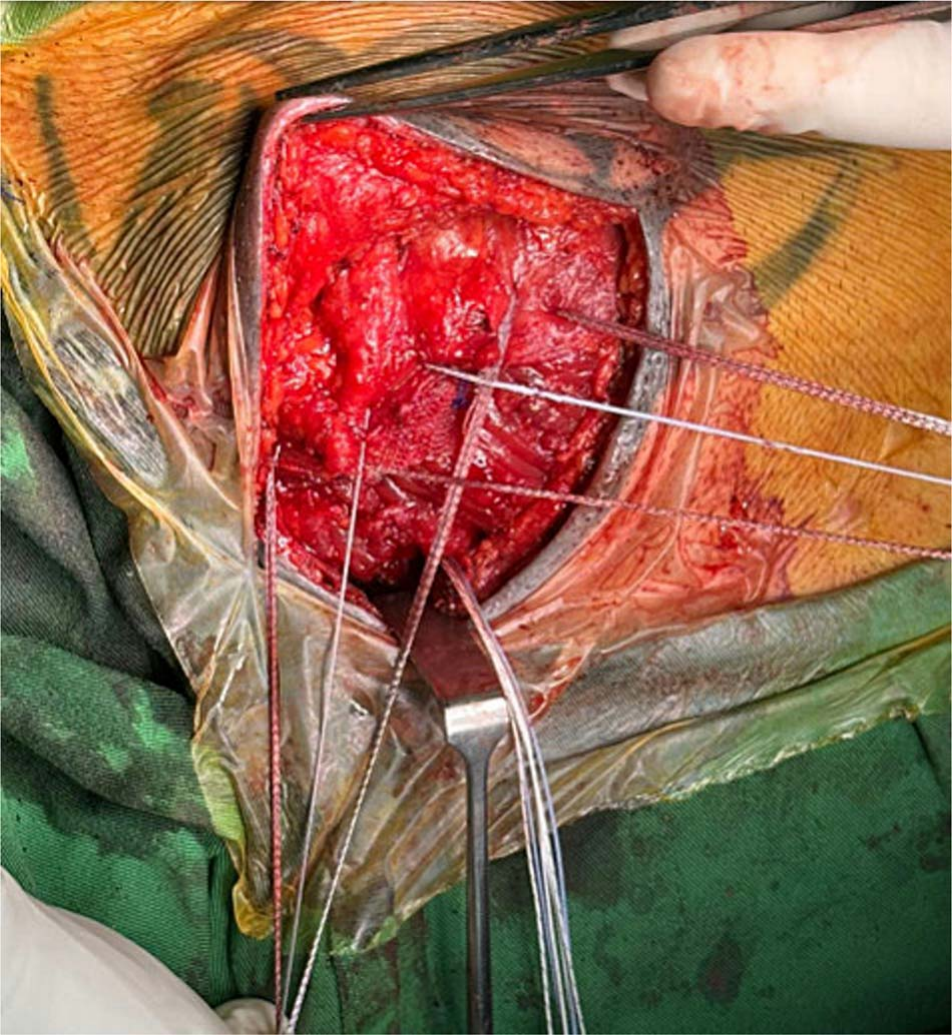
Intraoperative image showing placement of sutures through the mesh and PM muscle prior to securing them with sternal anchors.

**Figure 5:**
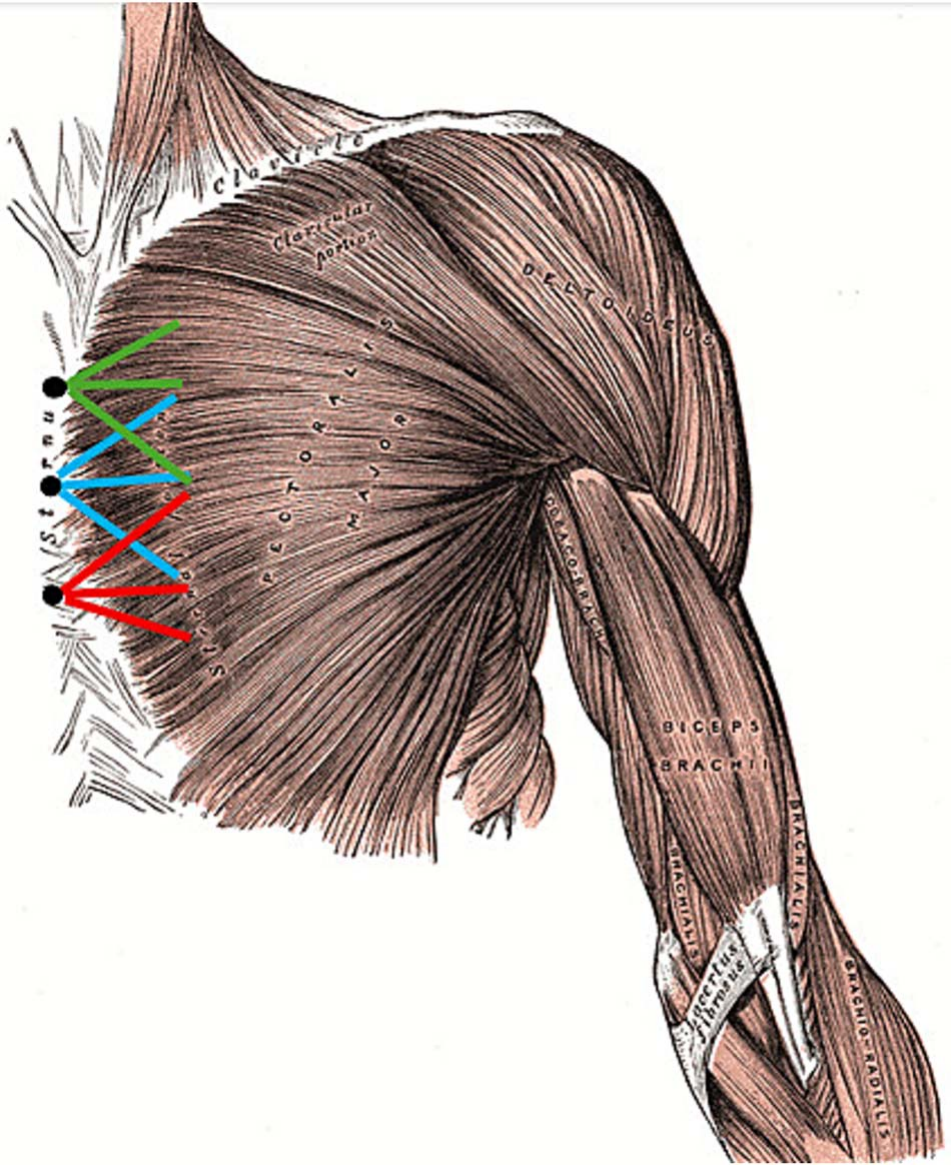
Anatomical representation of left PM repair using polypropylene mesh and knotless sternal anchors. Black dots: knotless anchor positions; blue lines: middle anchor sutures; green lines: proximal anchor sutures; red lines: distal anchor sutures. (Original public domain image by Henry Vandyke Carter, modified from *anatomy of the human body* by Henry Gray, 1918) [5].

The proximal anchor received three sutures, which were woven through the mesh and PM before being locked into the anchor, the other anchors received sutures in a similar fashion (Figs 4 and 5).

The construct was over-tensioned to compensate for expected postoperative laxity.

### Follow-up and outcomes

Immediate postoperative period: The patient experienced a rapid decrease in pain and was discharged on the first postoperative day with a shoulder immobilizer.

2 weeks: Wound healing progressed uneventfully, with no signs of infection or complications.

1 month: Physical examination revealed normal passive range of motion, restored muscle contouring at the sternum, and mild residual edema. Physical therapy was initiated.

6 months: Near-normal strength and near-full range of motion were achieved, confirming successful rehabilitation, and surgical repair.

## Discussion

The PM muscle, with its extensive clavicular, sternal, and costal origins, serves as a critical structure for upper extremity function. While Pâtissier first described this injury in 1822 [1], contemporary literature reports a marked increase in incidence, particularly among weight-training athletes [2,6].

El Maraghy *et al*. [6] established the foundational framework through their systematic review of 365 cases, developing a classification system incorporating timing, location, and tear characteristics. Their work revealed that only 0.8% of ruptures (n = 3) involved the muscle origin, making our sternal-origin case exceptionally rare [6].

The management paradigm was further refined by Bodendorfer *et al*. [4], whose meta-analysis confirmed superior outcomes with operative repair while establishing key principles: acute repairs (<6 weeks) achieve optimal results, chronic repairs require modified techniques [4]. However, most surgical outcome data focus is on humeral-side repairs, creating a knowledge gap regarding optimal management of rare origin avulsions – particularly in delayed presentations with significant retraction.

Our mesh-anchor technique addresses these challenges through several principles; first, the mesh can easily cover the broad origin of the PM. Second, having the mesh sutured to both the fascia and the muscle before being secured to the sternal anchors ensures proper and even distribution of the tensile forces acting on the muscle. Third, the porous structure of the mesh serves as a biological scaffold. Fourth, the 3-anchor-suture construct, as demonstrated in Fig. 5, provides the necessary strength and stability for proper healing and integration. Our success in managing this unique case further builds upon the findings of Al Abbassi *et al*. [3] that chronic tears, even with substantial retraction, can also benefit from surgical repair.

## Conclusion

This case report describes the successful application of a combined surgical mesh-suture-anchor reconstruction technique for a rare chronic sternal-origin PM rupture. Problems related to delayed repair were addressed: the extensive retraction (typical of chronic cases [7]), and the suboptimal tissue quality related to delayed presentation. To tackle these challenges, we employed a technique that builds upon the already established protocols of Merolla *et al*. [1] and Butt *et al*. [2] for the repair of PM ruptures.

Although we must stress that longer term follow up is necessary to fully assess the success and the durability of this surgical technique, our immediate short-term outcomes appear to be very promising in terms of strength, mobility, cosmesis, and patient satisfaction. This shows that surgical treatment is still a feasible option for the management of PM ruptures that are not within the acute repair window established by Bodendorfer *et al*. [4].

We must shed light on several implications from this case: first, it is necessary to expand the current classification systems [6] to better address rare and challenging presentations of PM ruptures which will in turn guide surgical decision making. Second, Future research should also focus on the comparison of the mesh-anchor construct to the more traditional techniques that employ tendon grafts as described by Merolla *et al*. [1] third, we should re-assess the outcomes of surgical management of chronic cases, owing to the promising short-term results of our unique case.
